# 3,4,5-Trimethoxy­benzohydrazidium chloride

**DOI:** 10.1107/S1600536808036301

**Published:** 2008-11-13

**Authors:** Aamer Saeed, Amara Mumtaz, Hummera Rafique, Kazuma Gotoh, Hiroyuki Ishida

**Affiliations:** a Department of Chemistry, Quaid-i-Azam University, Islamabad 45320, Pakistan; b Department of Chemistry, Faculty of Science, Okayama University, Okayama 700-8530, Japan

## Abstract

The title compound, C_10_H_15_N_2_O_4_^+^·Cl^−^, was obtained as an unexpected by-product during the synthesis of 1-[2-(substituted ar­yl)]-3-methyl­pyrazol-5-ones. The hydrazide group is essentially planar, with an r.s.m. deviation of 0.020 (2) Å, and is oriented at a dihedral angle of 30.52 (3)° with respect to the benzene ring. In the crystal structure, the cations and anions are linked through N—H⋯O and N—H⋯Cl hydrogen bonds, forming a mol­ecular tape running along the *b* axis.

## Related literature

For general background, see: Jin *et al.* (2006 ▶); Song *et al.* (2005 ▶); Yang *et al.* (2007 ▶). For a related structure, see: Zareef *et al.* (2006 ▶). For bond-length data, see: Allen *et al.* (1987 ▶).
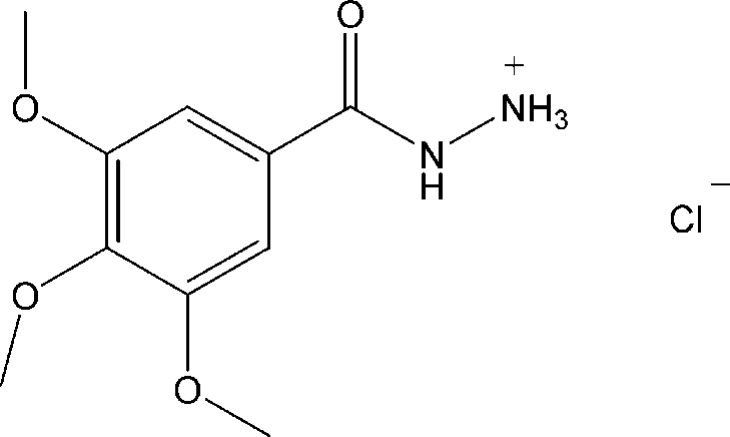


## Experimental

### 

#### Crystal data

C_10_H_15_N_2_O_4_^+^·Cl^−^
*M* *_r_* = 262.69
Monoclinic, 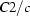

*a* = 38.587 (3) Å
*b* = 4.8202 (3) Å
*c* = 13.5915 (10) Å
β = 108.459 (2)°
*V* = 2397.9 (3) Å^3^
*Z* = 8
Mo *K*α radiation
μ = 0.32 mm^−1^
*T* = 223 (1) K
0.29 × 0.28 × 0.05 mm

#### Data collection

Rigaku R-AXIS RAPIDII diffractometer
Absorption correction: numerical (*ABSCOR*; Higashi, 1999 ▶) *T*_min_ = 0.930, *T*_max_ = 0.984
15536 measured reflections
3483 independent reflections
2987 reflections with *I* > 2σ(*I*)
*R*_int_ = 0.035

#### Refinement

*R*[*F* ^2^ > 2σ(*F* ^2^)] = 0.040
*wR*(*F* ^2^) = 0.111
*S* = 1.06
3483 reflections
158 parameters
H atoms treated by a mixture of independent and constrained refinement
Δρ_max_ = 0.51 e Å^−3^
Δρ_min_ = −0.19 e Å^−3^

### 

Data collection: *PROCESS-AUTO* (Rigaku/MSC, 2004 ▶); cell refinement: *PROCESS-AUTO*; data reduction: *CrystalStructure* (Rigaku/MSC, 2004 ▶); program(s) used to solve structure: *SHELXS97* (Sheldrick, 2008 ▶); program(s) used to refine structure: *SHELXL97* (Sheldrick, 2008 ▶); molecular graphics: *ORTEP-3* (Farrugia, 1997 ▶); software used to prepare material for publication: *CrystalStructure* and *PLATON* (Spek, 2003 ▶).

## Supplementary Material

Crystal structure: contains datablocks global, I. DOI: 10.1107/S1600536808036301/hk2567sup1.cif

Structure factors: contains datablocks I. DOI: 10.1107/S1600536808036301/hk2567Isup2.hkl

Additional supplementary materials:  crystallographic information; 3D view; checkCIF report

## Figures and Tables

**Table 1:** Hydrogen-bond geometry (Å, °)

*D*—H⋯*A*	*D*—H	H⋯*A*	*D*⋯*A*	*D*—H⋯*A*
N1—H1⋯O4^i^	0.876 (19)	2.007 (19)	2.8200 (13)	154.0 (18)
N2—H2*NA*⋯Cl1^ii^	0.90	2.25	3.1169 (11)	162
N2—H2*NB*⋯Cl1	0.90	2.20	3.0937 (11)	171
N2—H2*NC*⋯Cl1^iii^	0.90	2.30	3.1724 (12)	164

Symmetry codes: (i) 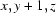; (ii) 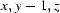; (iii) 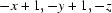.

**Fig. 1.**
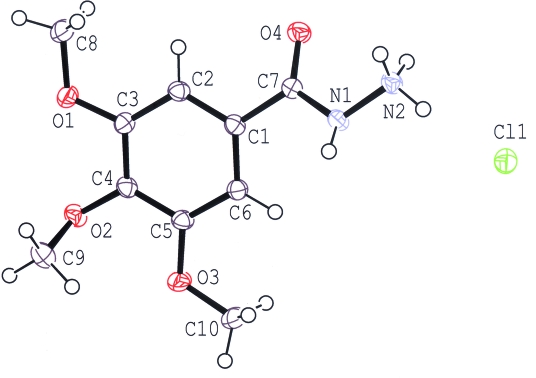
The molecular structure of the title molecule, with the atom-numbering scheme. Displacement ellipsoids are drawn at the 50% probability level.

**Fig. 2.**
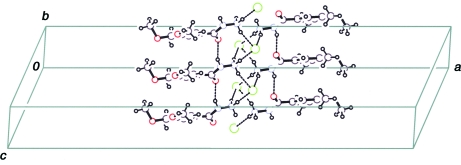
A partial packing diagram, showing the molecular tape running along the b axis. Hydrogen bonds are shown as dashed lines.

**Fig. 3.**
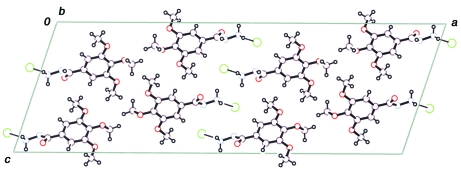
A crystal packing diagram, viewed along the b axis.

## supplementary crystallographic information

### Comment

3,4,5-Trimethoxybenzohydrazide is an intermediate toward variety of hetero- cyclic systems. Thioether derivatives bearing 1,3,4-thiadiazole and 3,4,5-tri- methoxyphenyl moieties and N-substituted benzylidene-3,4,5-trimethoxybenzo- hydrazide and 3-acetyl-2-substituted phenyl-5-(3,4,5-trimethoxyphenyl)-2,3 -dihydro-1,3,4-oxadiazole derivatives were proved to have good anti-cancer and anti-tumor bioactivities (Song *et al.*,  2005; Jin *et al.*, 2006). 4-Alkyl(aryl)- thioquinazoline derivatives synthesized from gallic acid were highly effective against cancer cell lines (Yang *et al.*,  2007). The title compound was obtained as an unexpected by-product during synthesis of 1-[2-(substituted aryl)]-3 -methylpyrazol-5-ones, and we report herein its crystal structure.

In the title compound (Fig. 1), the bond lengths (Allen *et al.*, 1987) and angles are within normal ranges, and may be compared with the corresponding ones in 3,4,5-trimethoxybenzohydrazide hemihydrate (Zareef *et al.*, 2006). The (N1/N2/O4/C7) plane is oriented with respect to ring A (C1-C6) at a dihedral angle of 30.52 (3)°, which is larger than the corresponding one [9.27 (10)°] in 3,4,5-trimethoxybenzohydrazide hemihydrate.

In the crystal structure, the molecules are linked through N-H···O and N-H···Cl hydrogen bonds, forming a molecular tape running along the b axis (Fig. 2). No significant interaction is observed between the tapes (Fig. 3).

### Experimental

A mixture of 3,4,5-trimethoxybenzohydrazide (0.01 mol) and ethyl acetoacetate (0.01 mol) was refluxed in methanol (25 ml), containing concentrated hydrochloric acid (1 ml) for 8 h in a water-bath. The resulting solution was then concentrated and cooled at room temperature. The solid thus separated was washed with methanol, dried and recrystallized with acetone. Anal. calcd. for C_10_H_15_Cl_N_2_O_4: C 45.72, H 5.76, N 10.66%; found: C 45.57, H 5.64, N 10.69%.

### Refinement

H1 atom (for NH) was located in difference synthesis and refined isotropically [N-H = 0.876 (19) Å and U_iso_(H) = 0.039 (5) Å^2^]. The remaining H atoms were positioned geometrically, with N-H = 0.90 Å (for NH_3_) and C-H = 0.94 and 0.97 Å for aromatic and metyl H, respectively, and constrained to ride on their parent atoms with U_iso_(H) = xU_eq_(C,N), where x = 1.2 for aromatic H and x = 1.5 for all other H atoms.

### Crystal data

**Table:**

C_10_H_15_N_2_O_4_^+^·Cl^–^	*F*_000_ = 1104.00
*M**_r_* = 262.69	*D*_x_ = 1.455 Mg m^−^^3^
Monoclinic, *C*2/*c*	Mo *K*α radiation λ = 0.71075 Å
Hall symbol: -C 2yc	Cell parameters from 12804 reflections
*a* = 38.587 (3) Å	θ = 3.0–30.0º
*b* = 4.8202 (3) Å	µ = 0.32 mm^−^^1^
*c* = 13.5915 (10) Å	*T* = 223 (1) K
β = 108.459 (2)º	Platelet, colorless
*V* = 2397.9 (3) Å^3^	0.29 × 0.28 × 0.05 mm
*Z* = 8	

### Data collection

**Table:**

Rigaku R-AXIS RAPIDII diffractometer	3483 independent reflections
Detector resolution: 10.00 pixels mm^-1^	2987 reflections with *I* > 2σ(*I*)
*T* = 223(2) K	*R*_int_ = 0.035
ω scans	θ_max_ = 30.0º
Absorption correction: numerical(ABSCOR; Higashi, 1999)	*h* = −54→50
*T*_min_ = 0.930, *T*_max_ = 0.984	*k* = −6→6
15536 measured reflections	*l* = −19→19

### Refinement

**Table:**

Refinement on *F*^2^	Secondary atom site location: difference Fourier map
Least-squares matrix: full	Hydrogen site location: inferred from neighbouring sites
*R*[*F*^2^ > 2σ(*F*^2^)] = 0.040	H atoms treated by a mixture of independent and constrained refinement
*wR*(*F*^2^) = 0.111	*w* = 1/[σ^2^(*F*_o_^2^) + (0.0672*P*)^2^ + 0.9082*P*] where *P* = (*F*_o_^2^ + 2*F*_c_^2^)/3
*S* = 1.06	(Δ/σ)_max_ = 0.003
3483 reflections	Δρ_max_ = 0.51 e Å^−^^3^
158 parameters	Δρ_min_ = −0.19 e Å^−^^3^
Primary atom site location: structure-invariant direct methods	Extinction correction: none

### Special details

**Table:**

Geometry. All e.s.d.'s (except the e.s.d. in the dihedral angle between two l.s. planes) are estimated using the full covariance matrix. The cell e.s.d.'s are taken into account individually in the estimation of e.s.d.'s in distances, angles and torsion angles; correlations between e.s.d.'s in cell parameters are only used when they are defined by crystal symmetry. An approximate (isotropic) treatment of cell e.s.d.'s is used for estimating e.s.d.'s involving l.s. planes.
Refinement. Refinement of *F*^2^ against ALL reflections. The weighted *R*-factor *wR* and goodness of fit *S* are based on *F*^2^, conventional *R*-factors *R* are based on *F*, with *F* set to zero for negative *F*^2^. The threshold expression of *F*^2^ > σ(*F*^2^) is used only for calculating *R*-factors(gt) *etc*. and is not relevant to the choice of reflections for refinement. *R*-factors based on *F*^2^ are statistically about twice as large as those based on *F*, and *R*- factors based on ALL data will be even larger.

### Fractional atomic coordinates and isotropic or equivalent isotropic displacement parameters (Å^2^)

**Table:**

	*x*	*y*	*z*	*U*_iso_*/*U*_eq_	
Cl1	0.531194 (8)	0.74543 (6)	0.14789 (2)	0.02591 (11)	
O1	0.29508 (2)	−0.1702 (2)	0.04400 (7)	0.0261 (2)	
O2	0.29359 (2)	0.12014 (18)	0.20875 (7)	0.02407 (19)	
O3	0.35072 (3)	0.4308 (2)	0.31676 (7)	0.0301 (2)	
O4	0.43237 (2)	−0.16714 (18)	0.07270 (7)	0.02515 (19)	
N1	0.44683 (3)	0.2704 (2)	0.12743 (9)	0.0220 (2)	
N2	0.47777 (3)	0.24854 (19)	0.09232 (9)	0.0222 (2)	
H2NA	0.4900	0.0916	0.1173	0.033*	
H2NB	0.4925	0.3956	0.1150	0.033*	
H2NC	0.4703	0.2446	0.0225	0.033*	
C1	0.38998 (3)	0.0818 (2)	0.13600 (9)	0.0198 (2)	
C2	0.35979 (3)	−0.0716 (2)	0.07763 (9)	0.0207 (2)	
H2	0.3616	−0.1909	0.0248	0.025*	
C3	0.32687 (3)	−0.0453 (2)	0.09891 (8)	0.0204 (2)	
C4	0.32509 (3)	0.1212 (2)	0.18165 (9)	0.0209 (2)	
C5	0.35556 (3)	0.2748 (2)	0.23870 (9)	0.0217 (2)	
C6	0.38830 (3)	0.2592 (2)	0.21544 (9)	0.0212 (2)	
H6	0.4087	0.3656	0.2524	0.025*	
C7	0.42422 (3)	0.0465 (2)	0.10930 (8)	0.0189 (2)	
C8	0.29543 (4)	−0.3376 (3)	−0.04212 (10)	0.0302 (3)	
H8A	0.3035	−0.2270	−0.0904	0.045*	
H8B	0.2710	−0.4073	−0.0767	0.045*	
H8C	0.3120	−0.4923	−0.0179	0.045*	
C9	0.27160 (4)	0.3649 (3)	0.17816 (11)	0.0297 (3)	
H9A	0.2862	0.5274	0.2066	0.045*	
H9B	0.2509	0.3552	0.2041	0.045*	
H9C	0.2628	0.3775	0.1031	0.045*	
C10	0.38005 (4)	0.6091 (3)	0.37142 (10)	0.0311 (3)	
H10A	0.4011	0.4981	0.4084	0.047*	
H10B	0.3726	0.7210	0.4205	0.047*	
H10C	0.3864	0.7294	0.3225	0.047*	
H1	0.4380 (5)	0.439 (4)	0.1231 (14)	0.039 (5)*	

### Atomic displacement parameters (Å^2^)

**Table:**

	*U*^11^	*U*^22^	*U*^33^	*U*^12^	*U*^13^	*U*^23^
Cl1	0.02223 (16)	0.02581 (17)	0.02881 (17)	−0.00046 (9)	0.00682 (12)	−0.00128 (10)
O1	0.0225 (4)	0.0335 (5)	0.0239 (4)	−0.0077 (3)	0.0095 (3)	−0.0079 (4)
O2	0.0240 (4)	0.0253 (4)	0.0279 (4)	−0.0016 (3)	0.0153 (3)	0.0007 (3)
O3	0.0292 (4)	0.0377 (5)	0.0280 (4)	−0.0087 (4)	0.0158 (4)	−0.0148 (4)
O4	0.0266 (4)	0.0200 (4)	0.0313 (5)	−0.0006 (3)	0.0127 (4)	−0.0037 (3)
N1	0.0220 (5)	0.0169 (5)	0.0317 (5)	−0.0004 (3)	0.0149 (4)	−0.0014 (4)
N2	0.0214 (5)	0.0202 (5)	0.0280 (5)	−0.0019 (3)	0.0122 (4)	−0.0009 (4)
C1	0.0209 (5)	0.0190 (5)	0.0213 (5)	0.0004 (4)	0.0092 (4)	0.0015 (4)
C2	0.0234 (5)	0.0199 (5)	0.0208 (5)	−0.0012 (4)	0.0099 (4)	−0.0010 (4)
C3	0.0218 (5)	0.0207 (5)	0.0195 (5)	−0.0028 (4)	0.0074 (4)	0.0005 (4)
C4	0.0216 (5)	0.0228 (5)	0.0213 (5)	−0.0010 (4)	0.0109 (4)	0.0008 (4)
C5	0.0246 (6)	0.0229 (5)	0.0199 (5)	−0.0010 (4)	0.0103 (4)	−0.0022 (4)
C6	0.0216 (5)	0.0215 (5)	0.0217 (5)	−0.0025 (4)	0.0086 (4)	−0.0020 (4)
C7	0.0202 (5)	0.0180 (5)	0.0186 (5)	0.0010 (4)	0.0062 (4)	0.0019 (4)
C8	0.0284 (6)	0.0371 (7)	0.0255 (6)	−0.0072 (5)	0.0092 (5)	−0.0091 (5)
C9	0.0275 (6)	0.0294 (6)	0.0358 (7)	0.0029 (5)	0.0152 (5)	0.0030 (5)
C10	0.0328 (6)	0.0344 (7)	0.0274 (6)	−0.0074 (5)	0.0112 (5)	−0.0118 (5)

### Geometric parameters (Å, °)

**Table:**

O1—C3	1.3581 (14)	C2—C3	1.3950 (15)
O1—C8	1.4251 (15)	C2—H2	0.9400
O2—C4	1.3771 (13)	C3—C4	1.4009 (15)
O2—C9	1.4356 (16)	C4—C5	1.3982 (16)
O3—C5	1.3605 (14)	C5—C6	1.3981 (16)
O3—C10	1.4280 (15)	C6—H6	0.9400
O4—C7	1.2271 (14)	C8—H8A	0.9700
N1—C7	1.3602 (14)	C8—H8B	0.9700
N1—N2	1.4230 (13)	C8—H8C	0.9700
N1—H1	0.876 (19)	C9—H9A	0.9700
N2—H2NA	0.9000	C9—H9B	0.9700
N2—H2NB	0.9000	C9—H9C	0.9700
N2—H2NC	0.9000	C10—H10A	0.9700
C1—C6	1.3947 (15)	C10—H10B	0.9700
C1—C2	1.3958 (15)	C10—H10C	0.9700
C1—C7	1.4867 (15)		
			
C3—O1—C8	117.43 (9)	C4—C5—C6	120.52 (10)
C4—O2—C9	114.20 (9)	C1—C6—C5	118.34 (10)
C5—O3—C10	117.16 (9)	C1—C6—H6	120.8
C7—N1—N2	116.00 (9)	C5—C6—H6	120.8
C7—N1—H1	120.7 (11)	O4—C7—N1	120.50 (10)
N2—N1—H1	113.3 (11)	O4—C7—C1	123.82 (10)
N1—N2—H2NA	109.5	N1—C7—C1	115.68 (10)
N1—N2—H2NB	109.5	O1—C8—H8A	109.5
H2NA—N2—H2NB	109.5	O1—C8—H8B	109.5
N1—N2—H2NC	109.5	H8A—C8—H8B	109.5
H2NA—N2—H2NC	109.5	O1—C8—H8C	109.5
H2NB—N2—H2NC	109.5	H8A—C8—H8C	109.5
C6—C1—C2	121.99 (10)	H8B—C8—H8C	109.5
C6—C1—C7	121.46 (10)	O2—C9—H9A	109.5
C2—C1—C7	116.55 (10)	O2—C9—H9B	109.5
C3—C2—C1	119.01 (10)	H9A—C9—H9B	109.5
C3—C2—H2	120.5	O2—C9—H9C	109.5
C1—C2—H2	120.5	H9A—C9—H9C	109.5
O1—C3—C2	124.68 (10)	H9B—C9—H9C	109.5
O1—C3—C4	115.46 (10)	O3—C10—H10A	109.5
C2—C3—C4	119.86 (10)	O3—C10—H10B	109.5
O2—C4—C5	120.82 (10)	H10A—C10—H10B	109.5
O2—C4—C3	118.97 (10)	O3—C10—H10C	109.5
C5—C4—C3	120.13 (10)	H10A—C10—H10C	109.5
O3—C5—C4	115.21 (10)	H10B—C10—H10C	109.5
O3—C5—C6	124.26 (11)		
			
C6—C1—C2—C3	0.46 (17)	O2—C4—C5—O3	−3.67 (16)
C7—C1—C2—C3	−179.61 (10)	C3—C4—C5—O3	179.53 (10)
C8—O1—C3—C2	−0.71 (17)	O2—C4—C5—C6	175.34 (10)
C8—O1—C3—C4	178.87 (11)	C3—C4—C5—C6	−1.46 (17)
C1—C2—C3—O1	175.98 (10)	C2—C1—C6—C5	2.13 (17)
C1—C2—C3—C4	−3.58 (16)	C7—C1—C6—C5	−177.79 (10)
C9—O2—C4—C5	77.58 (14)	O3—C5—C6—C1	177.30 (11)
C9—O2—C4—C3	−105.59 (12)	C4—C5—C6—C1	−1.62 (17)
O1—C3—C4—O2	7.64 (15)	N2—N1—C7—O4	5.88 (16)
C2—C3—C4—O2	−172.76 (10)	N2—N1—C7—C1	−173.78 (10)
O1—C3—C4—C5	−175.50 (10)	C6—C1—C7—O4	150.77 (11)
C2—C3—C4—C5	4.09 (17)	C2—C1—C7—O4	−29.15 (16)
C10—O3—C5—C4	−174.81 (11)	C6—C1—C7—N1	−29.58 (15)
C10—O3—C5—C6	6.22 (18)	C2—C1—C7—N1	150.50 (11)

### Hydrogen-bond geometry (Å, °)

**Table:**

*D*—H···*A*	*D*—H	H···*A*	*D*···*A*	*D*—H···*A*
N1—H1···O4^i^	0.876 (19)	2.007 (19)	2.8200 (13)	154.0 (18)
N2—H2NA···Cl1^ii^	0.90	2.25	3.1169 (11)	162
N2—H2NB···Cl1	0.90	2.20	3.0937 (11)	171
N2—H2NC···Cl1^iii^	0.90	2.30	3.1724 (12)	164

Symmetry codes: (i) *x*, *y*+1, *z*; (ii) *x*, *y*−1, *z*; (iii) −*x*+1, −*y*+1, −*z*.
